# Analysis of influencing factors of early neurological improvement after intravenous rt-PA thrombolysis in acute anterior circulation ischemic stroke

**DOI:** 10.3389/fneur.2022.1037663

**Published:** 2022-10-17

**Authors:** Zhang Xiufu, Liang Ruipeng, Zhou Jun, Li Yonglong, Wang Yulin, Zeng Jian, Chen Xianglin, Shen Lan, Zhang Zuowen

**Affiliations:** ^1^Department of Radiology, Chongqing University Jiangjin Hospital, Chongqing, China; ^2^Department of Neurology, Chongqing University Jiangjin Hospital, Chongqing, China

**Keywords:** acute ischemic stroke, early neurological improvement, intravenous recombinant tissue-type plasminogen activator, clinical factors, anterior circulation

## Abstract

**Background and objective:**

It has been widely reported that Early neurological improvement (ENI) after rt-PA intravenous thrombolysis contributes to a good long-term prognosis in patients experiencing acute ischemic stroke (AIS). However, which clinical factors influence after intravenous administration of recombinant tissue-type plasminogen activator (IV-rt PA) in AIS patients ENI is still unclear. This study aimed to evaluate the impact of influencing factors on the benefit of ENI after intravenous thrombolysis neurological improvement after IV-rt PA.

**Methods:**

The data of 73 patients with acute anterior circulation ischemic stroke who received intravenous thrombolysis with rt-PA in Chongqing University Jiangjin Hospital from January 2021 to July 2022 were retrospectively studied. According to the change rate of 24 h NISHH score, the research subjects were divided into the recovery group, the significant curative effect group, the curative effect group and the no curative effect group, the ENI after intravenous thrombolysis with rt-PA was defined as the improvement rate of National Institutes of Health Stroke Scale (NIHSS)score >46% at 24 h after IV-rt PA, and univariate factor analysis was used Clinical factors associated with ENI after intravenous thrombolysis.

**Results:**

According to the 24-h NIHSS improvement rate of rt-PA intravenous thrombolysis in patients with acute anterior circulation ischemic stroke, 35 cases (47.95%) of the study population had ENI. There was no statistical difference between the improvement and non-improvement group in general demographic data, stroke TOAST classification, stroke risk factors (history of stroke, heart disease, hyperlipidemia, hypertension), and laboratory test data. There was a statistically significant difference in the random blood glucose levels between the two groups (*p* < 0.001, *t* = 3.511).

**Conclusion:**

The effect of rt-PA intravenous thrombolysis within the time window of patients with acute anterior circulation ischemic stroke is significant, but the ENI after thrombolysis is easily affected by the level of blood glucose; diabetes is the most important factor affecting the acute anterior circulation ischemic stroke patients Clinical factors of ENI after intravenous thrombolysis with rt-PA.

## Introduction

Acute ischemic stroke (AIS) is currently one of the most common and serious diseases in the world and has become one of the leading causes of death and disability worldwide (1). Early vascular recanalization to restore normal blood flow perfusion is the most effective way to reduce disability and mortality in patients with AIS. Intravenous thrombolysis with recombinant tissue plasminogen activator (rt-PA) is the most economical and convenient treatment method (2). rt-PA is a serine protease synthesized by vascular endothelial cells, which selectively binds to plasminogen in thrombus. Numerous studies have demonstrated that patients with AIS benefit from intravenous thrombolysis.

However, the prognosis of stroke is still not optimistic despite the continuous progress in the treatment of stroke, and some stroke patients who have been actively treated remain severely disabled or even die. There is some uncertainty about the outcome of reperfusion therapy. Therefore, identifying clinical features that influence improved patient outcomes before initiating treatment may help to develop preventive measures in patients with established risk factors. This avoids ineffective vascular recanalization.

Early neurological deterioration (END) following rt-PA treatment of AIS is a serious clinical adverse event and leads to high mortality and poor neurological function (3). However, its specific pathogenesis is still unclear. In contrast, early neurological improvement (ENI) often predicts better thrombolysis and better long-term neurological function. The aim of this study was to investigate the clinical factors associated with ENI within 24 h in AIS patients receiving intravenous rt-PA. To evaluate the clinical risk factors of stroke patients who cannot benefit from intravenous thrombolytic therapy and provide clinical data for further basic medical research to clarify the self-stress protective mechanism after reperfusion therapy.

## Methods

### Patients information

Retrospective analysis of 127 patients with acute circulatory ischemic stroke who received intravenous thrombolysis with rt-PA in our hospital from January 2021 to July 2022, all patients have been diagnosed and have intravenous thrombolytic conditions. An onset-to-treatment time within 4.5 h after the stroke onset. Family members or patients signed the informed consent for intravenous thrombolysis.

Exclusion criteria: (1) younger than 18 years old or older than 85 years old; (2) patients undergoing thrombolytic therapy beyond the time window; (3) bridging patients who received rt-PA intravenous thrombolysis followed by mechanical thrombectomy; (4) Baseline NIHSS score of < 3 points; (5) Incomplete clinical data.

Collect general demographic and clinical data from all included populations, including sex, age, stroke risk factors (hypertension, hyperlipidemia, diabetes, coronary heart disease, atrial fibrillation, history of the previous stroke), TOAST typing, imaging data for leukoplasmic lesions, laboratory test data (liver function, renal function, blood count), baseline admission, 1 h thrombolysis, and 24 h National Institutes of Health Stroke Scale (NIHSS).

### Thrombolysis methods

All patients were treated with rt-PA intravenous thrombolytic (Boehringer, Ingelheim), a total of 0.9 mg/kg. The first 10% rt-PA is given in a 1 min intravenous bolus, and the remaining 90% is dissolved in normal saline and infused intravenously in 1 h.

### Definition of ENI

The NIHSS scale was used to evaluate patients for neurological deficits, and the higher the score, the more severe the neurological deficits. In this study, the NIHSS score at 24 h was first performed on the patients, and then the NIHSS change rate at 24 h was evaluated. The 24 h NIHSS change rate = (24 h NIHSS score-baseline NIHSS score)/baseline NIHSS score. The groups were set according to the 24 h NIHSS change rate, respectively: recovery: ①24 h NIHSS change rate ≥91%, ②significant curative effect: 46% ≤ 24 h NIHSS change rate < 91%, ③curative effect: 18% ≤ 24 h NIHSS change rate < 46%, ④no curative effect: 24 h NIHSS change rate < 18% or 24 h NIHSS score > baseline NIHSS score. ENI: NIHSS improvement rate ≥46% at 24 h.

### Statistical analysis

SPSS 22.0 and graph prism 9 software were used for data processing and chart making. First, the data were tested for normality. Measurement data that conformed to normal distribution were expressed as mean ± standard deviation ($\bar{X}\;\pm$ s), and a comparison between groups was performed using an Independent sample *t*-test; Measurement data with skewed distribution were expressed as the median and interquartile range (M, IQR), and comparisons between groups were performed using the Mann-Whitney *U*-test or the Wilcoxon matched-pairs signed rank test. The counting data is expressed in the number of cases (%), and the comparison is measured by χ2. The difference in *P* < 0.05 is statistically significant.

## Results

### General information

A retrospective analysis of stroke patients with baseline NIHSS score ≥ 3 who received rt-PA intravenous thrombolysis for acute anterior circulation ischemic stroke in our hospital from January 2021 to July 2022 127 cases. Among them, 17 patients were older than 85 years old, seven patients exceeded the 4.5 h rt-PA intravenous thrombolysis time window, and 30 patients underwent mechanical thrombectomy intervention immediately after receiving rt-PA intravenous thrombolysis. Finally, a total of 73 patients with acute anterior circulation ischemic stroke were included in this study. The patients were 42–85 years old, with an average age of (70.99 ± 9.70) years, including 38 males and 35 females.

**Figure 1:**
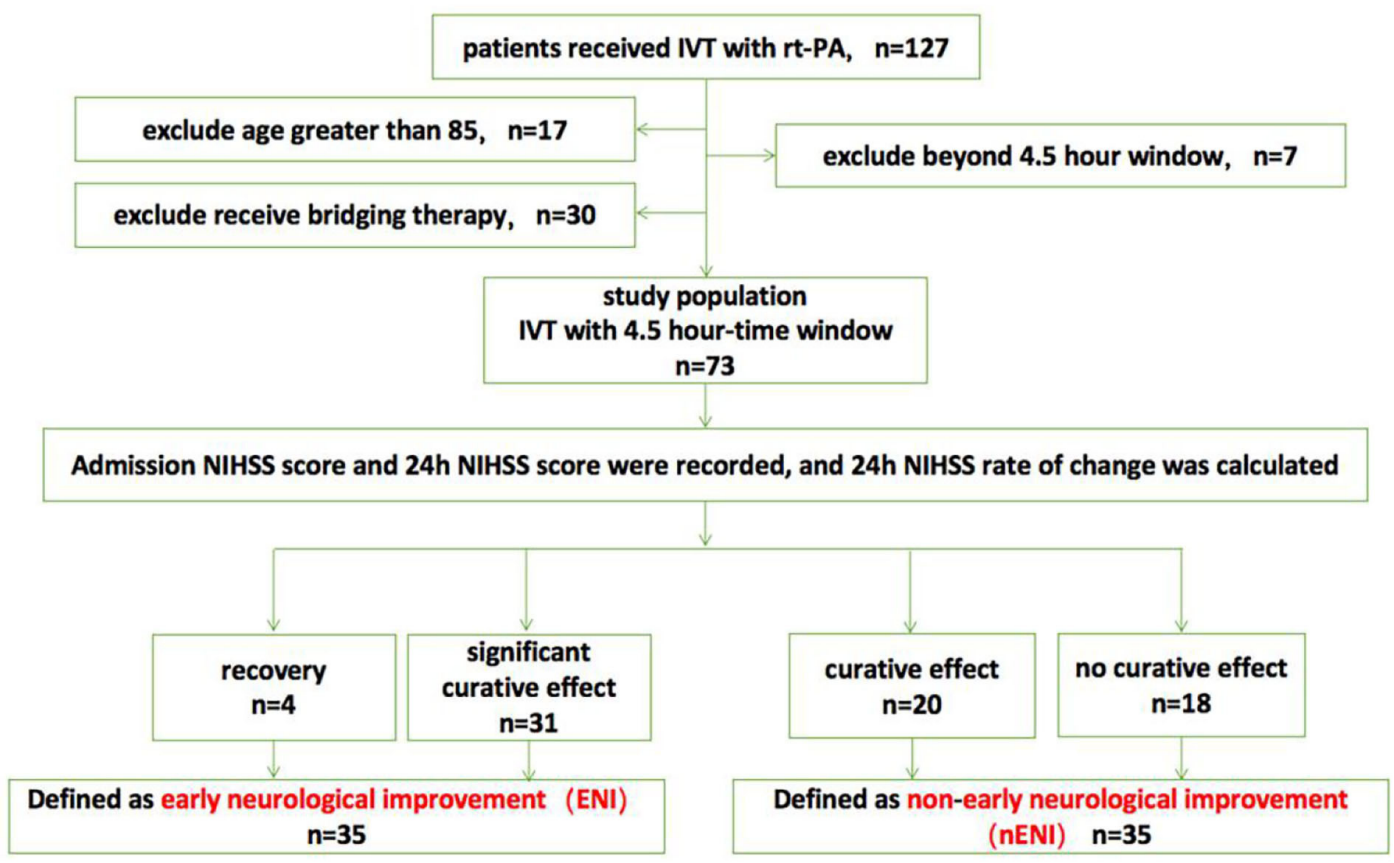
Flow chart of the study population. IVT, intravenous thrombolysis; NIHSS, National Institutes of Health Stroke Scale.

According to the 24-h NIHSS change rate, they were divided into recovery group (*n* = 4), significant curative effect group (*n* = 31), curative effect effective group (*n* = 20), and no curative effect group (*n* = 18). Among them, the recovery and the significant curative effect groups were defined as the ENI group after intravenous thrombolysis (*n* = 35), and the remaining two groups were defined as the non-early neurological benefit group after intravenous thrombolysis (*n* = 38) (Figure 1).

### Clinical effect of rt-PA intravenous thrombolysis in acute anterior circulation ischemic stroke within the time window

**Figure 2:**
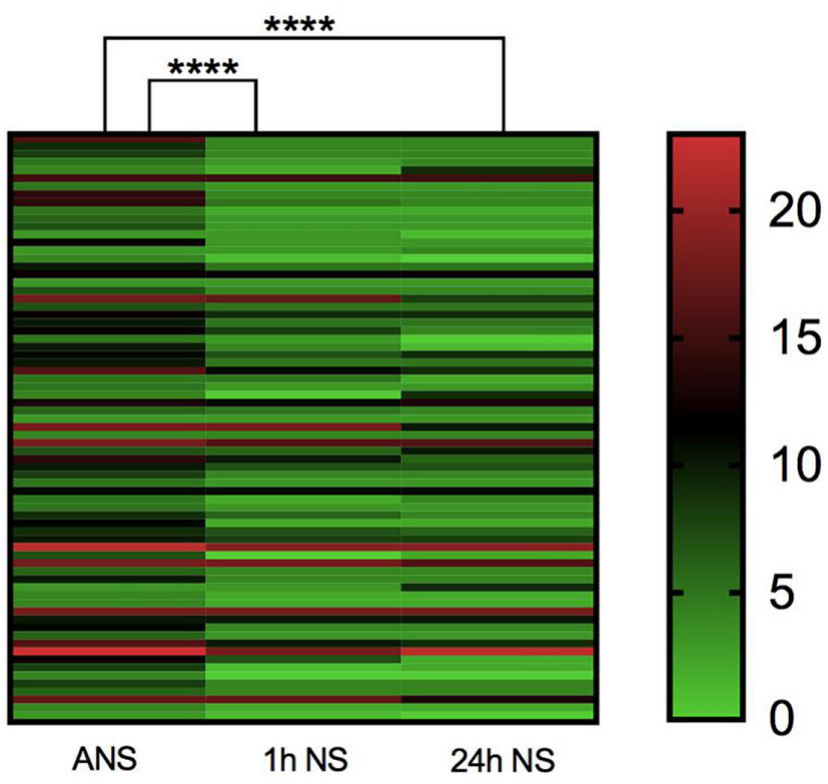
Heatmap of admission NIHSS score, 1 h NIHSS score of thrombolysis, and 24 h NIHSS score of thrombolysis. ****p < 0.0001.

As shown in Table 1, the median baseline NIHSS score on admission before thrombolysis was 8 (5–12) for all included study populations, and the median NIHSS score at 1 h after thrombolysis was 4 (3–8). There was a significant statistical difference between the two NIHSS scores (*p* < 0.0001, Z = −4.485). Among them, none of the patients had higher NIHSS scores 1 h after thrombolysis than before thrombolysis, and 12 patients had the same NIHSS score at 1 h after thrombolysis as the baseline NIHSS score at admission. The NIHSS score effective rate of 1 h thrombolysis was 71.23%. The median NIHSS score at 24 h after thrombolysis was 4 (3–9), which was significantly lower than the baseline NIHSS score at admission (*p* < 0.0001, Z = −4.714). Among them, the NIHSS score of five patients at 24 h after thrombolysis was higher than the baseline NIHSS score before thrombolysis, and the NIHSS score of six patients at 24 h after thrombolysis remained unchanged from the baseline NIHSS score at admission. The effective rate of rt-PA intravenous thrombolysis at 24 h was 75.40% (Table 1; Figure 2). In summary, rt-PA intravenous thrombolysis is effective in patients with acute precirculation ischemic stroke within the thrombolytic window.

**Table 1:** Admission NIHSS score, 1 h NIHSS score of thrombolysis, and 24 h NIHSS score of thrombolysis.

	**ANS (*n* = 73)**	**1 h NS (*n* = 73)**	**24 h NS (*n* = 73)**
NIHSS score (M, IQR)	8, 5–12	4, 3–8	4, 3–9
*Z*		−4.485	−4.714
*P*		< 0.0001	< 0.0001
Efficient		71.23%	75.4%

ANS, admission NIHSS score; 1 h NS, 1 h NIHSS score of thrombolysis; 24 h NS, 24 h NIHSS score of thrombolysis.

### Comparison of general demographic and clinical data

As shown in Table 2, univariate analysis showed that there were 13 patients with diabetes mellitus in the intravenous thrombolytic ENI group, accounting for 37.14%. There were 28 patients with diabetes mellitus in the non-early neurological benefit group, accounting for 73.68%. There was a statistically significant difference (*p* = 0.002, *t* = 9.881). The blood glucose level in the ENI group (6.19 ± 1.24) was significantly lower than that the non-early neurological benefit group (7.90 ± 2.71), and the difference was statistically significant (*p* < 0.001, *t* = 3.511). However, the two groups had no statistically significant differences in blood pressure, blood lipids, TOAST classification of stroke, coronary heart disease, atrial fibrillation, OTT, and white matter lesions. Therefore, this study found that stroke patients with diabetes mellitus and high fasting blood glucose level were not effective in early thrombolysis, and high blood glucose level was a negative factor affecting ENI after intravenous thrombolysis in patients with AIS.

**Table 2:** Baseline characteristics of subgroups based on the presence of post-thrombolysis ENI.

**Variable**	**Improvement group (*n* = 35)**	**Non-Improvement group (*n* = 38)**	***t*/*Z*/χ^2^**	***p***
Men (*n*, %)	21, 60.00%	17, 44.74%	1.701	0.192
Age (years, $\bar{X}$ ± s)	69.66 ± 11.25	72.21 ± 7.98	1.108	0.272
Hypertension (*n*, %)	25, 71.43%	34, 89.47%	3.827	0.074
SBP (mmHg, $\bar{X}$ ± s)	142.80 ± 14.29	143.82 ± 15.43	0.293	0.770
DBP (mmHg, $\bar{X}$ ± s)	81.23 ± 9.29	82.16 ± 8.83	0.438	0.663
Diabetes (*n*, %)	13, 37.14%	28, 73.68%	9.881	0.002*
RBG (mmol/L, $\bar{X}$ ± s)	6.19 ± 1.24	7.90 ± 2.71	3.511	< 0.001*
CHD (*n*, %)	10, 28.57%	13, 34.21%	0.269	0.604
Atrial fibrillation (*n*, %)	11, 31.43%	13, 34.21%	0.064	0.853
Prior stroke (*n*, %)	6, 17.14%	10, 26.32%	0.896	0.344
TOAST (*n*, %)			1.028	0.795
LAA	18, 51.43%	23, 60.53%		
CE	4, 11.43%	5, 13.16%		
SAO	12, 34.29%	9, 23.68%		
ODC	1, 2.86%	1, 2.63%		
Hyperlipidemia (*n*, %)	14, 40.00%	14, 36.84%	0.077	0.782
Leukocyte (10^9^/L, $\bar{M}$, IQR)	6.69 (5.48–8.15)	7.64 (5.83–9.95)	−1.717	0.086
Neutrophils (10^9^/L, $\bar{M}$, IQR)	5.06 (3.36–6.21)	5.32 (4.22–8.32)	−1.574	0.116
Lymphocytes (10^9^/L, $\bar{M}$, IQR)	1.13 (0.86–1.7)	1.17 (0.97–1.52)	−0.497	0.619
NLR (M, IQR)	4.02 (2.72–6.36)	4.05 (3.08–4.05)	−0.740	0.459
Uric acid (M, IQR)	329 (257–379)	294 (264–393)	−0.226	0.821
Cystatin C (mg/L, $\bar{M}$, IQR)	0.8 (0.71–0.96)	0.76 (0.67–1.00)	−0.624	0.533
Baseline NIHSS score ($\bar{M}$, IQR)	8 (5–11)	7 (5–13)	−0.476	0.634
OTT (*n*, %)			0.059	0.953
≤ 3 h	26, 74.29%	28, 73.68%		
>3 h	9, 25.71%	10, 26.32%		
WML Fazekas ($\bar{M}$, IQR)	3 (2–4)	2 (2–4)	−0.046	0.963
PVL ($\bar{M}$, IQR)	1 (1–2)	1 (1–2)	−0.110	0.912
DMVL ($\bar{M}$, IQR)	1 (1–2)	1 (1–2)	0	1

SBP, Systolic blood pressure; DBP, Diastolic blood pressure; RBG, Random blood glucose; CHD, Coronary heart disease; LAA, Large-artery atherosclerosis; CE, Cardioembolism; SAO, Small-artery occlusion; SOD, Stroke of other determined cause; NLR, neutrophil to lymphocyte ratio; WML, White Matter Lesion; PVL, periventricular leukomalacia; DWML, deep white matter lesions.

### Comparison of blood glucose levels between the recovery, significant curative effect, curative effect and no curative effect groups

The random blood glucose data of the recovery group, the significant curative effect group, the curative effect group and the no curative effect group were selected for verification and comparison to further clarify the correlation between the random blood glucose level and the short-term neurological improvement of patients after rt-PA intravenous thrombolysis.Since there were few cases in the recovery group, only 4 patients, they were included in the significant curative effect group (*n* = 35). Considering the large fluctuation of blood glucose levels after stroke, the Mann Whitney *U*-test was used to test blood glucose between groups. The median value of the blood glucose level was compared between the significant curative effect group, the effective group and the no curative effect group. The results are shown in Table 3, and the random blood glucose levels in the significant curative effect group are significantly lower than those in the effective group and the no curative effect group (*p* = 0.01, *Z* = −2.563; *p* = 0.002, *Z* = 3.08). These results indicate that the level of blood glucose affects the short-term improvement of neurological function in stroke patients after intravenous thrombolysis with rt-PA.

**Table 3:** Comparison of random blood glucose levels between groups.

	**RSCE (*n* = 35)**	**CE (*n* = 20)**	**NCE (*n* = 18)**
RBG (mmol/L, M, IQR)	6.03 (5.02–7.01)	6.95 (6.00–9.30)	7.31 (6.38–8.26)
*Z*		−2.563	−3.080
*P*		0.01	0.002

RSCE, recovery and significant curative effect; CE, curative effect; NCE, no curative effect.

## Discussion

This retrospective study aimed to investigate the clinical factors affecting ENI in patients with acute anterior circulation ischemic stroke after intravenous thrombolysis with rt-PA and shows that diabetes mellitus (hyperglycemic levels) is a clinical risk factor for ENI after thrombolysis. In the comparison of blood glucose levels of the recovery group, the significant curative effect group, the curative effect group and the no curative effect group after rt-PA intravenous thrombolysis therapy, it was found that the blood glucose level showed a gradually increasing trend with the decrease of the improvement rate of the NIHSS score at 24 h, which further clarified the predictive value of higher blood glucose levels for the early neurological dysfunction results of patients after rt-PA intravenous thrombolysis. This study indicates that patients with high RBG levels should be monitored more carefully.

Previous studies have shown a large number of clinical factors influencing the early efficacy of thrombolysis, among which intravenous thrombolytic ENI (or END) is associated with many factors such as age, sex, history of heart disease, stroke history, level of inflammatory factors, diabetes mellitus, leukoplasmic lesions, site of infarction, and area of infarction (4–7). A statistical analysis of the above risk factors was included in this study and found a statistically significant difference between the glycemic factor alone and the ENI after rt-PA thrombolysis. No other factors were found to have a significant correlation with ENI after rt-PA intravenous thrombolysis. It is speculated that the reason may be the differences in the grouping criteria of this study and other studies and the different definitions of ENI (benefit or worsening) after rt-PA intravenous thrombolysis in acute stroke. For example, Wang Ling et al. defined 24 h NIHSS score ≥4 as early neurological deterioration (8). Kim et al. defined 24 h NIHSS score ≥2 as early neurological deterioration (9). The European cooperative acute stroke study (ECASS)-I define a score of 24 h in the scandinavian neurological stroke scale (SSS) that reflects the level of consciousness or motor function ≥ 2 points or a decrease in language function score from baseline≥3 as an early deterioration in neurological function (10). The rate of change in NIHSS scores is more objectively indicative of the changes in neurological function before and after thrombolysis in stroke patients, and Kharitonova et al. (11). NiHSS scores improve by 40% on baseline or NIHSS scores of 0–1 after thrombolysis as the evaluation method for ENI. However, we did not define ENI by grouping according to the above criteria. In this study, we excluded patient data with a score of < 3 and selected the NIHSS score at 24 h to improve by 46% or more from the baseline as the objective evaluation standard to define ENI.

Excluding glycemic factors, no general statistics and stroke risk factors were found to influence post-rt-PA thrombolytic ENI in stroke patients. Our findings are consistent with previous findings that patients with severe stress hyperglycemia have a higher risk of developing END after IV-rt PA, confirming that blood glucose is a marker of poor prognosis in patients undergoing intravenous thrombolytic therapy for ischemic stroke (8). Ribo et al. (12) studied that the acute increase in blood glucose after stroke would hinder the process of fibrinolysis and affect the blood reperfusion in the ischemic penumbra. A study suggested that initial blood glucose level may be an independent risk factor for END (13).

An in-depth understanding of how hyperglycemia levels and imbalances in insulin regulation mechanisms act on the rt-PA intravenous thrombolytic process and thus affect the benefits of postvenous thrombolytic nerve function in patients with AIS with diabetes mellitus is critical. Previous studies have reported that acute ischemia and hypoxia in stroke cause neurocellular oxygen glucose deprivation, ATP depletion, sodium and potassium pump imbalance, excitatory amino acid production in large quantities, intracellular calcium overload, and free radical elevation, which in turn leads to neuronal cell death. Elaborating on how the above pathophysiological changes and hyperglycemia levels regulate the influence of the rt-PA thrombolysis mechanism in terms of signaling mechanism helps to find the target of drug intervention and the timely application of drug intervention before and after thrombolysis is extremely important to improve stroke prognosis. First, after acute stroke, brain tissue ischemia and hypoxia will convert aerobic respiration into anaerobic glycolysis, while increased blood sugar will aggravate the process of anaerobic glycolysis, increase the accumulation of lactic acid in nerve tissue and further promote the transformation of asymptomatic neural tissue into symptomatic neural tissue (14). Secondly, the stress on the body after cerebral ischemia further increases the blood sugar level. The underlying mechanism of the impact of stress hyperglycemia on early neurological function may involve a greater inflammatory response and neurohormonal response, which aggravates brain edema and lactic acid accumulation, induces increased apoptosis and increases oxidative stress in BBB endothelial cells. Finally, the extensive microangiopathies caused by diabetes lead to poor regulation and tolerance of blood pressure fluctuations. The reduced compensatory ability of collateral circulation leads to reduced oxygen supply to brain tissue and local metabolic disorders, which eventually aggravates brain edema and the production of oxygen free radicals. Further leads to malignant brain edema and hemorrhagic transformation (15). In addition, an imbalance of the coagulation/fibrinolytic system is critical for the formation and progression of thrombus and for recurrent thrombus formation. Increased glucagon secretion and insulin resistance *in vivo* also affect the efficacy of rt-PA intravenous thrombolysis. The combination of rt-PA and urokinase, the main activators of the plasma fibrinolytic system, leads to a decrease in fibrinolytic activity. Hyperglycemia disrupts the balance of coagulation and fibrinolytic mechanisms by influencing endothelial cell function (16). The main causes affecting ENI are symptomatic intracranial hemorrhage (sICH), malignant edema. In this study, there were few cases of complications. The patients only showed mild oral mucosa and gingival bleeding, and there were no cases of severe intracranial hemorrhage and malignant edema. This may be related to the strict screening of intravenous thrombolytic indications to maximize the exclusion of people with complications or to the exclusion from the study cohort in cases of potential complications followed by mechanical thrombolytic intervention. In short, diabetes can mediate neuronal cell damage and apoptosis through various channels, and the above-related pathophysiological mechanisms are discussed to further explore and establish targets for mid-stroke drug intervention.

Glycemic factors are clear and important interventional risk factors for stroke and prognosis, and it is critical to delve into the mechanisms by which high glycemic levels and insulin affect the benefits of thrombolytic nerve function. Importantly, ischemia, oxidative stress, inflammation, hemorrhagic transformation, malignant edema and herniation, timing issues of neurological deterioration and how diabetes intervenes in the above procedures are the next issues that require focused research.

In addition, the four cured cases in the retrospective study (24 h NIHSS change rate ≥91%) all belonged to the arteriolar occlusion type in the TOAST classification, indicating the early prognosis of this type of stroke was good. However, no difference in TOAST typing was observed in the univariate analysis of ENI, and further long-term follow-up studies still needed to be compared.

Our study also has many limitations: (1) This study is a single-center retrospective study; (2) The sample size is small; (3) The patient's long-term prognosis results were not collected; (4) Blood glucose levels are detected as random blood glucose. In future studies, the sample size should be further expanded, the fasting blood glucose should be uniformly measured, and studies should be conducted on the relationship between early functional improvement and long-term clinical outcomes.

## Conclusion

ENI after intravenous thrombolysis with rt-PA in AIS patients is easily affected by the blood glucose level. Diabetes is a clinical factor affecting ENI after intravenous thrombolysis with rt-PA in patients with AIS.

## Data availability statement

The original contributions presented in the study are included in the article/supplementary material, further inquiries can be directed to the corresponding author.

## Ethics statement

Ethical review and approval was not required in accordance with local legislations. All patients signed the informed consent before receiving intravenous thrombolysis, and were informed that the data results would be used for research analysis, and any character with the subject's identity would be removed from the study results to ensure that personal privacy would not be disclosed and there would be no risk to the subjects.

## Author contributions

ZX, ZZ, and LR conceived and designed the study. LR wrote the manuscript and performed the statistical analysis. LY, ZJu, ZJi, SL, and CX collected data and organized the database. ZZ and ZX reviewed and edited the manuscript. ZX contributed to data curation and supervision. All authors read and approved the manuscript.

## Funding

This work was supported by the Science and Technology Plan Project of Chongqing Jiangjin Science and Technology Bureau, project No. 2022015.

## Conflict of interest

The authors declare that the research was conducted in the absence of any commercial or financial relationships that could be construed as a potential conflict of interest.

## Publisher's note

All claims expressed in this article are solely those of the authors and do not necessarily represent those of their affiliated organizations, or those of the publisher, the editors and the reviewers. Any product that may be evaluated in this article, or claim that may be made by its manufacturer, is not guaranteed or endorsed by the publisher.
